# Behavioral Problems In The Utilization Of New Technology To Control Caries: Patients And Provider Readiness And Motivation

**DOI:** 10.1186/1472-6831-6-S1-S5

**Published:** 2006-06-15

**Authors:** Philip Weinstein

**Affiliations:** 1 Department of Dental Public Health Sciences, University of Washington, Seattle, WA 98195, USA

## Abstract

New research developments frequently are neither adopted by providers nor utilized by patients. This dual problem of impacting the behaviors of providers and patients presents a challenge. This paper will present behavioral theories and technologies that can be utilized to impact both provider and patient behaviors.

## Introduction

### Provider Readiness

There is a very small literature on the efficacy of interventions impacting the behaviors of dental providers. The literature in medicine is larger, but also without much of a theoretical base [1].

Kanouse and Jacoby [2] suggest that the diffusion model [3] has led to an overemphasis on the importance of information, ignoring other factors such as the practitioners' motivation to change. Grimshaw and colleagues [4] suggest exploring the application of behavioral theories to the understanding of professional behavioral change. Similarly, Walker and colleagues [5] suggest the problem of understanding why health professionals do or do not change their practices is similar to finding out why people do or do not adopt a healthy lifestyle, a research area that has been extensively investigated. Interestingly, Parker and Parikh [6] hypothesize that health practitioner behaviors may not change as a consequence of continuing education because the learner is not "ready to change." These authors recommend Prochaska and DiClemente's [7] Transtheoretical Stages of Change Model. This model postulates that change is a process that proceeds through specific stages, each of which has key characteristics – characteristics that are amenable to targeted, stage-specific or matched interventions. The Transtheoretical Stages of Change Model may provide a theoretical model to guide the development and testing of provider interventions.

The Stages of Change Model has enjoyed considerable success as a means of understanding and promoting behavior change in people with addictive disorders and other unhealthful behaviors. This model has recently been applied to dental disorders [8-10]. The number of proposed stages and their definitions vary somewhat depending on the study, but five stages are generally cited as follows:

Precontemplation (not thinking about or planning to change within the next 6 months – What problem?)

Contemplation (considering, intending to change with in the next 6 months – Should I change?)

Preparation/Action (planning to change in the next month – Can I change?)

Action (making relevant changes – How do I change?)

Maintenance (sustaining change – Is it worth it?).

There is some evidence that the Stages of Change model is useful in understanding and influencing practitioner behaviors. Problem-oriented interventions may be useful. For example, Nilsson and colleagues [11] found feedback of individual prescribing rates combined with problem-oriented outreach visits effective in altering prescribing rates and patterns for hypertension. Intent to change, theoretically at the Preparation Stage, has been found to be associated with changes in managing cardiovascular disease [12]. Enhancing commitment to change can be seen as an intervention in its own right [13,14].

### Patient Readiness And Motivation

It is not surprising that information and education have been insufficient to alter the behaviors of the parents of children at risk for dental caries. While some parents of children with ECC are unaware of the etiology of this disease [15-17], research does not support the efficacy of providing information to the parents or caretakers [18-20]. Educating patients in dental and medical settings is frequently an exercise in overt persuasion. What appears to be a convincing line of reasoning to the dental professional falls on deaf ears or results in reluctance to change. Patients have reservations about "being told what to do" [21]. More fundamental is the possibility that direct persuasion, whatever the degree of readiness to change, pushes the patient into a defensive position. There is evidence that the effectiveness of advice giving (information with persuasion) about lifestyle change is only 5–10% [22].

While advice giving has not been successful, there have been promising results using a counseling approach, which is more patient-centered. For example, in dentistry a counseling approach has been found to be successful. Harrison and Wong [23] reported that children whose mothers had at least two counseling sessions had significantly fewer decayed surfaces than children at baseline. The counseling approach featured one-on-one counseling by a lay worker, personalization of recommendations, and telephone follow-up of mothers.

While the Stages of Change theory provides understanding of the process of change, Motivational Interviewing (MI), a brief counseling approach that focuses on skills needed to motivate others, provides strategies to move patients from inaction to action. This brief approach has been successful in impacting addictive behaviors and has recently been used to establish positive health-related behaviors [24]. Recently, Weinstein, Harrison and Benton [25,26] have reported a study of 240 high-risk infants aged 6 to 18 months and their parents. They were randomly assigned to MI or traditional health education groups. After two years there was a 50% reduction in the incidence of caries in the MI group.

The MI approach allows exploration of a problem in a supportive environment that expresses acceptance and provides affirmations of the person's strengths. It involves asking questions before providing information and advice. Persons are encouraged to talk, and there is an attempt to understand the person's frame of reference. These techniques are borrowed from Rogers' non-directive patient-centered therapy. However, the approach is directive; advice is given, with the person's permission, and is accompanied with encouragement to make choices.

There are two phases to MI. The client or patient is active in both phases. First there is an attempt to establish rapport and trust and to help identify the problem of concern. During this phase the patient or client moves from the Precontemplative to the Contemplative stage. In psychological field theory terms, the intent is to increase the gradient of approach. The goals are achieved primarily by asking open-ended questions and by demonstrating that the listener has heard the person by paraphrasing or summarizing (active listening). For example, in our protocol with the parents of 6–18 month old high-risk children, we asked the parent to answer the question, "What is it like to be Georgie's mom?" The next question focused on oral health. "Tell me about your dental health and the health of your family?" This was following by "What do you want for your child's dental health?" or "If I (or God) could grant you one wish for your child's teeth (a dental miracle), what would it be? The last question "sets the hook." The parent is now telling us what she desires for the oral health of her child.

The second phase involves moving from the Contemplative to the Preparation/Action stage. Patients at the Contemplative stage are said to be ambivalent; they have a need to change, but they perceive obstacles to change. The person is asked to weigh the pros and cons of changing. "What are the costs, the benefits of changing? What happens if you do nothing?" Choice is stressed, and there is discussion of the potential obstacles to action for each action option. Working with the person focuses mainly on identifying a plan to act. Menus of potential changes are used in even briefer versions of MI.

The attempt in this phase is to lower the gradient of avoidance. Behavior change technology is very useful in helping the transition between planning and sustained action. The emphasis in this technology is on the establishment and maintenance of habits. Continuity of care and follow-up is also part of this approach. A workbook useful in MI training for dental personnel has been written [27].

MI can be useful in helping establish and then maintain positive oral health behaviors. However, regimens that require considerable parental or patient attention will have higher failure rates. For example, taking a daily fluoride tablet has been found to be a problem for low-income persons [28]. Compliance with daily parental tooth cleaning and the chewing or ingestion multiple times a day of xylitol products may also be problematic. The more we require, the less the cooperation, even with an effective behavioral intervention. New dental interventions are needed that require as little as possible of patients.

While MI has not been used in changing provider behaviors, there is some evidence that a provider-centered approach has been successful. For example, Casebeer and colleagues [29] found that Web-based instruction for physicians that used (1) office-practice data to assess needs and (2) tailored case vignettes with feedback resulted in changes when compared to a control condition. Assessing the readiness of a dental provider to change existing practices and utilize new technology may prove to be a key in designing MI-driven interventions that may be used on the internet. Does the provider perceive limitations of the existing approach? Is he/she aware of the benefits of the new technology? What are the perceived problems in adapting the new technology? What happens if nothing is changed?

Although internet-based continuing education is still in its infancy, analyses of 16 controlled studies have provided subjective evidence of short-term changes in provider behaviors [30]. Moreover, MI software has been developed that is interactive and has been used on-line to tailor a counseling approach to identify and then reduce problem drinking [31]. The application of Stages of Change Theory and MI-like approaches has significant promise to impact the behaviors of both patients *and *providers.

## Competing interests

The author(s) declare that they have no competing interests.
